# Sexual behaviours and sexual health among middle-aged and older adults in Britain

**DOI:** 10.1136/sextrans-2021-055346

**Published:** 2022-08-11

**Authors:** Junead Khan, Emily Greaves, Clare Tanton, Hannah Kuper, Thomas Shakespeare, Eneyi Kpokiri, Yun Wang, Jason J Ong, Suzanne Day, Stephen W Pan, Weiming Tang, Bingyi Wang, Xin Peng, Bowen Liang, Huachun Zou, Joseph D Tucker, Dan Wu

**Affiliations:** 1 Department of Clinical Research, London School of Hygiene and Tropical Medicine, London, UK; 2 Department of Infectious Disease Epidemiology, London School of Hygiene and Tropical Medicine, London, UK; 3 Department of Population Health, London School of Hygiene and Tropical Medicine, London, UK; 4 Department of Pharmaceutical Economics and Policy, School of Pharmacy, Chapman University, Orange, California, USA; 5 Central Clinical School, Monash University, Carlton, Victoria, Australia; 6 Institute for Global Health and Infectious Diseases, University of North Carolina at Chapel Hill, Chapel Hill, North Carolina, USA; 7 Department of Health and Environmental Sciences, Xi'an Jiaotong-Liverpool University, Suzhou, Jiangsu, China; 8 UNC Project-China, Guangzhou, Guangdong province, China; 9 School of Public Health (Shenzhen), Sun Yat-Sen University, Shenzhen, Guangdong, China; 10 Kirby Institute, University of New South Wales, Sydney, New South Wales, Australia; 11 Department of Clinical Research, London School of Hygiene and Tropical Medicine Faculty of Infectious and Tropical Diseases, London, UK; 12 Department of Epidemiology, West China School of Public Health, Sichuan University, Chengdu, Sichuan, China

**Keywords:** Sexual Behavior, SEXUAL HEALTH, Sexual Dysfunction, Physiological

## Abstract

**Objectives:**

Population-representative studies of the sexual health of middle-aged and older adults are lacking in ageing societies. This study aimed to identify latent patterns of sexual behaviours and health of people aged 45–74 years.

**Methods:**

We conducted a latent class analysis of the National Attitudes and Sexual Lifestyles Survey (Natsal-3), a nationally representative survey conducted in Britain in 2011.

**Results:**

Of the 5260 respondents aged 45–74 years, 48.86% of men and 44.91% of women belonged to the Content Caseys class who reported good sexual health. The Infrequent Indigos (30.94% of men, 44.38% of women) were characterised by a lack of sexual activity, reported some dissatisfaction, and were more likely to have a disability. The Low-Functioning Lees (11.65% of men, 8.41% of women) reported some more disability and had issues with sexual functioning and higher levels of distress. The Multiple-Partnered Morgans (8.62% of men, 2.30% of women) were characterised by a greater number of sexual partners and several risk behaviours.

**Conclusions:**

The use of these four classes can aid in improved targeting of tailored sexual health services to improve sexual function, sexual satisfaction, reduce distress and risky behaviours among middle-aged and older adults. These services should be inclusive of the disabled community.

**WHAT IS ALREADY KNOWN ON THIS TOPIC**

- Population-representative sexual health studies often focused on young populations and few included middle-aged and older adults.

**WHAT THIS STUDY ADDS**

- Our latent class analysis using a nationally representative sample identified four different categories of sexual health needs among middle-aged and older adults in Britain.

**HOW THIS STUDY MIGHT AFFECT RESEARCH, PRACTICE AND/OR POLICY**

- Our study findings can help inform tailored clinical capacity training programmes to better meet the sexual health needs of middle-aged and older adults.

## Introduction

Longer life spans and increased quality of care for older people have contributed to a fundamental transition about what it means to be an older adult, altering sexual biographies and behaviours.^1 2^ Although sexual activity generally decreases with age,^3^ the English Longitudinal Study of Ageing showed that a sizeable portion of middle-aged and older men and women remain sexually active.^4^ However, there is a persistent misperception that ‘age is a condom’ and older adults aged ≥65 years do not have sex or sexual health needs.^5^ Additionally, research on sexual health among middle-aged adults has been limited,^6^ and many sexual health studies have either explicitly excluded older participants^7 8^ or aggregate entire subsets of middle-aged and older adults.^9 10^ There are few population-representative studies of sexual health that include these groups, even though this information is important for planning messaging campaigns, behavioural interventions, clinical programmes and health systems.^11^ There is therefore a large evidence gap on the sexual health needs of middle-aged and older adults, which themselves are heterogeneous across age groups and experience a range of sexual health needs.^11^

Previous studies have reported high levels of sexual dysfunction and dissatisfaction among older adults, where commonly reported conditions concern erectile problems for men, arousal for women and a general decline of interest in sex.^4 12^ In addition, sexually transmitted infections (STIs) are increasing among the older age groups,^13^ and few older adults report speaking to their doctor about sex at all.^12^ Thus the limited evidence we do have on older adults suggests a need to better identify and respond to older adults’ needs for sexual health services in ways that address their unique age-related concerns. Characterising these concerns and identifying the range of need for this diverse demographic will improve the ability of care providers and policymakers to prioritise interventions.

We sought to evaluate the sexual behaviours and health outcomes of middle-aged and older adults and identify sexual health needs among this group. Using the National Survey of Sexual Attitudes and Lifestyles (Natsal-3), a rare example of a population-representative sexual health study that included older adults, we conducted a latent class analysis (LCA) to create and examine identifiable classes. LCAs have recently gained attention through their ability to characterise differing levels of risk^14^ and health outcomes, including in sexual health.^15^ Past LCAs that cluster symptoms and biomarkers to study disease profiles offer a model for translating demographic and behavioural characteristics into clinical guides that could push forward a personalised approach to sexual health service provision.^16^ The purpose of this LCA subanalysis was to characterise the sexual health of this population and parse out differing sets of sexual health needs that can be used to develop and tailor appropriate services and interventions.

## Methods

### Overview

This study is a secondary analysis of data from Natsal-3, a nationally representative probability sample survey conducted in Britain in 2010–12.^17^ We included middle-aged people (ie, people aged 45–64 years, a period of human adulthood that immediately precedes the onset of old age) and older adults aged ≥65 years. We broadened our scope to include middle-aged adults because the 40s are associated with a transition from early to older adulthood in British life and culture and important biological changes (eg, female and male menopause). Other studies focusing on ageing have frequently included participants in their 40s and 50s.^18 19^

We used LCAs to identify unobserved (ie, latent) classes of middle-aged and older people with respect to their sexual health characteristics using sexual health items.^20^ This method uses responses to survey questions in order to identify new subgroups that are defined by observed variables. We then compared sociodemographic, health-related and help-seeking variables across classes. In doing this we hope to capture a better understanding of the population structure of middle-aged and older adults, the diversity of their experiences and needs, and to inform potential strategies for intervention.

### Participants and procedures

Natsal-3 was the third cross-sectional roughly decennial nationwide population-representative household survey focused on sexual lifestyle and attitudes in Britain. The survey recruited 15 162 men and women aged 16–74 years in 2010–2012. Natsal-3 is one of the first of its kind to survey older adults with this breadth and comprehensiveness and the next survey (Natsal-4), which is planned to be fielded in Spring 2022, will extend only up to 59 years of age.^21^ Methods describing the design and administration of Natsal-3 have been published previously.^22^

### Survey instruments and definitions

The survey asked participants for sociodemographic information. Participants were also asked about their opinions towards their general health status, treatment history for depression in the past 12 months, sexual behaviours and practices, sexual function, feeling of sexual satisfaction, sexual distress and history of STIs. For the purpose of this analysis we have defined ‘unsafe sex’ as the incidence of unprotected sex with two or more partners in the past year. ‘Low sexual function’ was derived from a 5-point Likert scale developed and validated by the Natsal-3 study team.^23^ Additionally, participants were also asked to identify whether they had a limiting disability, defined as any long-standing illness, disability or infirmity that limits their activities in any way. These questions are shown in online supplemental table I.

10.1136/sextrans-2021-055346.supp1 Supplementary data

### Statistical analysis

Analyses used complex survey functions in order to incorporate the weighting, clustering and stratification of the data. Weights were derived to account for unequal probabilities of selection and non-response and corrected for differences in gender, age and regional distribution according to the UK 2011 census for this analysis.^22^ We first reported differences in sociodemographic characteristics, sexual behaviours and outcomes for the entire age range of the sample using χ^2^ tests. We then focused our comparison on the 45–54, 55–64 and 65–74 age groups. Analyses were carried out using JMP Pro Version 15.0 (SAS Institute, Cary, North Carolina, USA).

### Latent class analyses (LCAs)

We hypothesised that men and women may show varying patterns of sexual practices and outcomes, and we stratified the LCAs by sex. We included a set of variables that were key measurements of sexual behaviour (ie, frequency of sex in last 4 weeks, number of partners in past year), higher-risk sexual behaviours (unsafe sex in past year, purchasing sex in past year) and sexual health outcomes (STI diagnosis in the past 5 years, sexual function, sexual satisfaction and sexual distress). Each latent class model item was coded as a binary variable to improve interpretability of model results. Identification of variables for inclusion was an iterative process, and the final list of variables was determined based on discussions among the authors and published literature. We determined the optimal number of classes based on Bayesian Information Criteria (BIC) (see more details in online supplemental table III and the descriptions).^24^ After identifying latent classes, we cross-tabulated on sociodemographic, behavioural and lifestyle variables by classes to investigate the individuals who make up the classes for potential tailoring of interventions.

## Results

### Demographic backgrounds

Data from 9902 respondents were eligible for this subanalysis. Among them, 5260 respondents aged 45–74 years were the focus of this study, of which 2233 were men and 3027 were women. Table 1 shows the demographic characteristics of participants alongside data for those aged <45 years. Among those aged 45–74 years, most identified as white (92% for both men and women) and over half (55% of men, 51% of women) were married or in a civil partnership. In addition, 28% of men and 30% of women had a limiting disability.

**Table 1:** Demographic characteristics of male and female participants in Natsal-3 by age group, 2012, UK (n=15 162)

Class label	Men (%)				Women (%)			
	<45*	45–54	55–64	65–74	<45*	45–54	55–64	65–74
	(n=4060)	(n=794)	(n=772)	(n=667)	(n=5842)	(n=1123)	(n=1030)	(n=874)
Ethnicity								
White	85	91	95	96	85	89	94	96
Mixed	3	1.0	0.5	0.6	3	2	0.9	0.3
Asian	7	4	2	2	6	3	3	1
Black	4	3	1	1	4	5	1	1
Other†	1.6	0.8	0.5	0.0	1.6	0.9	1	0.2
Sexual identity								
Heterosexual/straight	97	96	97	99	96	97	99	99
Gay/lesbian	2	2	1	0.2	1	1	0.8	0.1
Bisexual	1	1	1	0.5	2	1	0.0	0.3
Other	0.4	0.7	0.4	0.3	0.3	0.7	0.2	0.2
Relationship status								
Married or civil partnership	23	50	54	62	28	49	55	50
Living with a partner	18	12	7	3	20	9	5	3
In a steady relationship, not cohabiting	19	11	9	4	20	13	5	2
No steady relationship	39	24	27	26	31	27	33	41
Academic qualifications								
No academic qualifications	10	17	37	52	8	17	38	35
Academic qualifications typically gained at age 16 years‡	32	40	25	18	32	40	33	27
Studying for or have attained further academic qualifications	52	42	37	29	54	41	27	18
Socioeconomic class§								
Manager/professional	32	44	41	26	31	39	31	15
Intermediate	34	30	31	29	27	25	29	20
Semi-routine/routine	34	30	31	29	27	25	29	20
No job currently	3	4	9	27	8	11	18	50
Student	16	1	0.0	0.4	16	0.9	0.3	0.2
Respondent has a longstanding illness, disability or infirmity								
None	81	62	45	42	78	60	49	41
Non-limiting	9	16	24	25	11	13	20	26
Limiting	10	22	31	32	11	27	31	33
Opinion of own health								
Very good	46	35	26	24	46	36	32	25
Good	43	45	42	41	42	42	39	41
Fair	10	16	22	28	10	16	20	24
Bad	1	4	7	7	2	5	7	7
Very bad	0.4	0.8	3	1	0.3	1	2	2
Quintile of Index of Multiple Deprivation								
1 (least deprived)	18	23	22	26	17	23	23	25
2	19	22	25	26	19	21	23	25
3	19	19	21	19	20	20	20	18
4	23	18	16	17	22	17	20	16
5 (most deprived)	21	19	17	12	22	20	14	17

All numbers are weighted.

*Participants aged >16 years.

†Other includes Chinese.

‡English General Certificate of Secondary Education or equivalent.

§National Statistics Socio-Economic Classification.

### Behavioural characteristics

Online supplemental table II shows the behavioural characteristics of participants aged >45 years. Of these respondents, fewer men reported no sexual partners in the past year (27% of men, 37% of women) and more men reported having two or more partners in the past year (10% of men, 3% of women). Men also reported more often engaging in all other sexual practices with the exception of masturbation in the past month, which was more commonly reported by women (50% of men, 60% of women). Men were more commonly engaged in risky sexual behaviours such as unsafe sex in the past year (5% of men, 2% of women). Similar trends were observed for those diagnosed with an STI in the last 5 years (1% of men, 0.6% of women), while 17% of men and 16% of women reported a low sexual function score. All p values for this table were found to be significant.

**Table 2:** Latent class analysis response probabilities for men aged ≥45 years (n=1887)

Class label	Class 1	Class 2	Class 3	Class 4
	Content Caseys (%)	Infrequent Indigos (%)	Low-Functioning Lees (%)	Multiple-Partner Morgans (%)
	(n=913)	(n=583)	(n=218)	(n=173)
Overall class size	48.86	30.94	11.65	8.62
No sex in last 4 weeks*	16.63	**99.39**	42.48	25.51
Had unsafe sex in past year*	0.01	0.01	0.12	**68.60**
≥2 sex partners in past year*	1.24	0.02	5.93	**99.89**
Diagnosed with any STI in the last 5 years	0.78	0.02	2.34	4.30
Paid for sex in past year*	0.14	0.51	1.71	6.43
Low sexual function score	4.55	5.63	**98.81**	17.17
Dissatisfied with sex life	4.81	32.16	**53.78**	9.34
Distressed or worried about sex life	1.13	12.19	**57.40**	7.42

All estimates are unweighted.

Probabilities >50% are shown in bold type to indicate items that members of a given class were more likely to report. In naming and characterising the classes, dimensions in which classes differed strongly and a probability of >50% for a certain item were considered an indication that members of a given class were more likely to report that risk factor.

The methodology we employed presupposes the existence of discrete latent classes. However, there may be individuals who better fit one or more continuous latent dimensions. The model output expresses the probability of class membership for all individuals for each class as well as the probability of each characteristic given membership of each class. Given each individual had a particular probability of class membership for each class, individuals were assigned to the latent class for which they had the greatest probability of membership. Each class was named after the item that was most prevalent in members of that class.

*Same and opposite sex.

### Latent class analysis (LCA)

LCAs of sexual health-related variables explored several models that ranged from two to five latent classes with model fit statistics shown in online supplemental table III. We determined that the four-class model provided the optimal fit for both men and women.

**Table 3:** Latent class analysis response probabilities for women aged ≥45 years (n=2653)

Class label	Class 1	Class 2	Class 3	Class 4
	Content Caseys (%)	Infrequent Indigos (%)	Low-Functioning Lees (%)	Multiple-Partner Morgans (%)
	(n=1245)	(n=1144)	(n=206)	(n=58)
Overall class size	44.91	44.38	8.41	2.30
No sex in last 4 weeks*	23.02	**99.98**	45.72	41.28
Had unsafe sex in past year*	0.01	0.01	0.04	1.74
≥2 sex partners in past year*	0.91	0.01	1.65	**99.76**
Diagnosed with any STI in the last 5 years	0.30	0.65	0.18	6.27
Paid for sex in past year*	0.01	0.10	0.50	0.20
Low sexual function score	6.67	6.36	**96.17**	37.13
Dissatisfied with sex life	0.61	20.73	**66.81**	30.55
Distressed or worried about sex life	2.69	6.90	**55.49**	11.46

All estimates are unweighted.

Probabilities >50% are in bold type to indicate items that members of a given class were more likely to report.

*Same and opposite sex.

The results in tables 2 and 3 show the conditional probabilities of reporting a behaviour given membership in a certain class for both men and women, respectively. With the intent to provide simple and useful characterisations of the latent classes, we termed these four subgroups using common gender-neutral names in the UK and their primary descriptors. Alliterative gender-neutral class names were chosen to reduce negative connotations with stigmatised characteristics. These four names might aid clinicians in identifying sexual health needs and identifying services in a manner to which each could be most receptive and most relevant. These probabilities formed the basis for the labelling of each class as follows. The first and largest class, made up of 48.86% of the men and 44.91% of the women, was labelled ‘Content Caseys’ based on the low likelihoods of reporting higher-risk sexual behaviours, multiple partners and dissatisfaction/distress. Class 2, a smaller class for men (30.94%) than women (44.38%), was labelled ‘Infrequent Indigos’ based on the high likelihood that members of this class had not engaged in sex in the last 4 weeks. Class 3, made up of 11.65% of the men and 8.41% of the women, was labelled ‘Low-Functioning Lees’ based on the greater probability that members of this class reported a low sexual function score as well as distress regarding their sex life. The fourth class, made up of more men (8.62%) than women (2.30%), was labelled ‘Multiple Partner Morgans’ as a result of their high likelihood of having ≥2 sexual partners in the past year.

### Demographic, health and lifestyle characteristics by classes

The results in table 4 show the percentages reporting a given sociodemographic, general health or help-seeking characteristic given membership in a certain latent class. Owing to the similar trends observed among men and women in the creation of these classes, we combined both sexes for this analysis to aid in readability and simplicity. Separate analyses are reported in online supplemental tables IV and V.

**Table 4:** Sociodemographic, health and lifestyle characteristics by latent classes of Natsal-3 participants aged ≥45 years (n=4540)

Class label	Class 1	Class 2	Class 3	Class 4	P value
	Content Caseys (%)	Infrequent Indigos (%)	Low-Functioning Lees (%)	Multiple-Partner Morgans (%)	
	(n=2158)	(n=1727)	(n=424)	(n=231)	
Age group					<0.0001
45–54	47	21	46	56	
55–64	34	35	35	34	
65–74	20	45	19	10	
Ethnicity					0.12
White	94	95	94	92	
Mixed	0.9	0.6	0.9	1	
Asian	3	2	3	1	
Black	2	2	2	5	
Other	0.5	0.7	0.2	0.9	
Relationship status					<0.0001
Married or civil partnership	72	34	71	26	
Living with a partner	9	3	9	5	
Steady relationship, not cohabiting	13	0.7	7	22	
No steady relationship	7	63	13	48	
Education					<0 .0001
No academic qualifications	28	43	27	33	
Academic qualifications typically gained at age 16 years*	37	28	34	37	
Studying for or have attained further academic qualifications	34	27	38	30	
Quintile of Index of Multiple Deprivation					<0.0001
1 (least deprived)	26	19	25	20	
2	25	22	19	21	
3	19	21	19	19	
4	16	19	17	20	
5 (most deprived)	14	20	20	20	
Sought help or advice for sex life in the past year					<0.0001
Yes	7	7	32	13	
No	93	94	68	87	
Currently taking medicine prescribed by a doctor for depression					<0.0001
Yes	5	10	13	10	
No	95	90	87	90	
Medications in last year that have limited sexual activity or enjoyment					<0.0001
Yes	7	9	23	11	
No	93	88	77	89	
Respondent’s opinion of own health					<0.0001
Very good	37	25	25	31	
Good	45	38	41	42	
Fair	15	26	26	20	
Bad	4	9	7	6	
Very bad	0.6	2.9	1.9	0.9	
Respondent has a longstanding illness, disability or infirmity					<0.0001
None	59	44	46	60	
Non-limiting	20	21	20	16	
Limiting	21	35	34	25	

All estimates are unweighted.

*English General Certificate of Secondary Education or equivalent.

Broadly, the Content Caseys and Low-Functioning Lees were very similar in profile. In the Low-Functioning Lees class, the majority were married or cohabitating with a partner (71% and 9%, respectively) while, conversely, the majority of the Infrequent Indigos were in no steady relationship (63%). The Multiple-Partner Morgans class contained a higher percentage of those in no relationship (48%) than those who were married (26%) and, of the four classes, the largest share of those who were in a relationship without cohabitating (22%). Help-seeking for one’s sexual health was more common for the Low-Functioning Lees (32%) and the Multiple-Partner Morgans (13%) than for those in the other two classes. Regarding disability, the Infrequent Indigos and Low-Functioning Lees classes had a higher proportion of individuals reporting limiting disabilities (35% and 34%) than the Content Caseys and Multiple-Partner Morgans (21% and 25%).

## Discussion

Population-representative studies that focus on sexual practices, lifestyles and outcomes among middle-aged and older adults are rare. Our analyses extend the literature by examining sexual lifestyle and outcomes and identifying latent classes among middle-aged and older adults in Britain. Overall, about half of all middle-aged and older adults were Content Caseys who had good sexual health and reported few issues concerning their sex lives. The other three classes suggested unique needs for sexual health services. A large proportion of these were Infrequent Indigos who reported no recent sexual activity, the vast majority of whom are single. Smaller proportions of survey respondents reported issues with sexual function (Low-Functioning Lees) and/or multiple partners (Multiple-Partner Morgans). Factors such as relationship status, disability and behaviour are likely important in determining the type of need or risk at hand.

The Low-Functioning Lees reported the highest levels of distress about their sex lives, as well as higher levels of dissatisfaction and a relatively high need for professional advice compared with other classes. Because Low-Functioning Lees were associated with more distress, we speculate that these tensions can be compounded among those cohabiting with a partner. This is unlike the Infrequent Indigos, who cohabitated much less and largely did not report much distress, and the Content Caseys who had a similar relationship profile but reported less distress and higher sexual function scores. In addition to helping improve sexual function, interventions to reduce this psychological stress for Low-Functioning Lees, particularly those in long-term relationships, will be essential in taking a comprehensive approach of sexual health for this class. One study analysing high-need patients at a federally qualified health centre in the USA found that their LCA model helped the centre recommend new patients to existing health services.^25^ Using a similar approach, referrals to psychological services, relationship counselling and even sex therapy involving both partners could improve outcomes for this class.^4 26^

As indicated by the class descriptions of the Low-Functioning Lees and Infrequent Indigos, we noticed the concurrence of reporting a disability that limited one’s activities and the reporting of sexual function issues and/or infrequent sex. These classes also observed issues with dissatisfaction and distress and more from these groups tend to have poorer general health and more disability. These findings on disability and dysfunction are consistent with previous studies which reported on the interrelated nature of general and mental health and sexual well-being.^18 27^ Due to these higher levels of distress and dissatisfaction, there is likely a missed opportunity to tailor sexual health services for these two classes. With Infrequent Indigos and Low-Functioning Lees, our findings suggest that sexual health and well-being should be managed alongside general health and that doctors should proactively assess sexual functioning and sexual lifestyle concerns within the management of disability care. As with LCA-guided referrals, our model can help identify need and raise notice to sexual health issues in clinical sessions that may otherwise be missed, particularly among non-sexual health physicians.^28^ Our hope is that the model can serve as a useful heuristic in these more generalised non-sexual health settings for the complexity and interconnectivity of these sexual health issues.

Individuals in the Multiple-Partner Morgan class were more likely to have recent risky sexual behaviours and to report a recent STI diagnosis. Combined with their relatively low rates of help-seeking, these behaviours and health history suggest an elevated STI burden among this class and the potential usefulness for STI education services. Many older adults came of age prior to the HIV crisis and the proliferation of modern sexual health education campaigns, and therefore did not receive these services as adults nor as adolescents.^28–30^ Those in the Multiple-Partner Morgans class could potentially benefit from provider-initiated conversations about safer sex, STI prevention and STI testing during routine clinical visits. Our LCA results informed that clinicians may find it useful to engage in capacity planning for this class to help identify and streamline these patients.^25^ Next steps for research would include the training and validation of a multiclass model based on the latent groups shown here, with the hopes of informing predictions of class membership as probabilities based on real-time patient data.^31^ This processing of building and validating a model would require many more data than are available here, and we suggest that next steps make efforts to include anonymized clinical data.

Beyond the clinic, these four classes can be used to gain detailed insight into public health campaigns. LCAs can be used to tailor mass education campaigns, community engagement initiatives or clinical interventions among the different classes.^32^ As this LCA contributes to the research by identifying sets of sexual health needs and areas for more personalised intervention, the potential for the use of these new subgroups for analysis, both as a model in small-scale clinical tailoring and of use in broader intervention research, should be further explored.

### Limitations

Our use of Natsal-3 survey data does have some limitations, most notably the age of the data as interviews were conducted a decade ago (2010–2012). While these data are now old, they are not necessarily outdated as the relative lack of attention to older adults in sexual health research and the ageing of the British population suggests the relevance of our analysis today. Although some items such as online dating have likely changed since 2012, the English Longitudinal Study of Ageing noted in 2016 comparable levels of sexual activity and dissatisfaction to Natsal-3,^4^ suggesting minimal changes in sexual behaviour of older adults in recent years.

The original survey did not collect information on intersex individuals, transgender individuals or individuals identifying as a non-binary gender. Although gender identity is now recognised as a meaningful part of sexual well-being and our data likely include these individuals, we report no findings specific to these demographics. Also, the data do not represent those individuals in institutionalised living such as care homes, in which predisposing demographics and frequent lack of sexual health policies^25^ could mean an elevated risk of dysfunction or STIs. In addition, the Natsal-3 study did not survey respondents aged >74 years, thus rendering a clear yet incomplete picture of older adults who can remain sexually active well into their 80s and beyond.^4^

## Conclusions

Overall, older adults have sex late into their lives and, while many maintain good sexual health, about half experience issues with either low sexual function, distress about sex or an increased risk for STIs. These reports include those with disabilities. Identifying specific sets of needs and potential areas of risk through our class models can aid researchers and clinicians in tailoring and improving sexual health services to various subgroups of an ageing population.

10.1136/sextrans-2021-055346.supp2 Abstract translation This web only file has been produced by the BMJ Publishing Group from an electronic file supplied by the author(s) and has not been edited for content.

## Data Availability

Data are available in a public, open access repository.
