# Supplementary material for: Sexual behaviours and sexual health among middle-aged and older adults in Britain
Source: Sex Transm Infect. 2022 Aug 11;99(3):173–9. doi: 10.1136/sextrans-2021-055346 (PMC10176408; doi:10.1136/sextrans-2021-055346)
Supplement: Supplementary data [file sextrans-2021-055346supp001.pdf]

Appendix Table I. Definitions of key variables used in the analysis and how they were measured

Variable	Definition of Variable	How variable was asked in Natsal-3
Relationship Status	Married or civil partnership if their response was 1 or 2<br>Living with partner if their response was 3<br>In steady relationship, not cohabitating if their response was 4<br>No steady relationship if their response was 5	At present are you...<br>1. Married and living with your (husband/wife)<br>2. In a registered same-sex civil partnership and living with your partner<br>3. Living with a partner, as a couple (not married or in a civil partnership)<br>4. In a steady relationship, but not living together<br>5. None of the above
Ethnicity	White if their response was A: 1, 2, or 3<br>Mixed if their responses was B: 4, 5, 6, or 7<br>Asian if their response was C: 8, 9, 10, or 11<br>Black if their response was D: 12, 13, or 14<br>Chinese if their response was E: 15<br>Other if their response was E:16	Participants were asked about which ethnic group they belong to. They could choose:<br>**A. White**<br>1. British<br>2. Irish<br>3. Any Other White background<br>**B. Mixed**<br>4. White and Black Caribbean<br>5. White and Black African<br>6. White and Asian<br>7. Any Other Mixed background<br>**C. Asian or Asian British**<br>8. Indian<br>9. Pakistani<br>10. Bangladeshi<br>11. Any Other Asian background<br>**D. Black or British Black**<br>12. Caribbean<br>13. African<br>14. Any Other Black background<br>**E. Chinese or other ethnic group**<br>15. Chinese<br>16. Any Other
Sexual Identity	Participants were asked to choose from a list sexual orientations on a card and indicate the one that best fits how they think of themselves.	Participants were asked to choose how they best identify themselves. Their options were:<br>1. Heterosexual / Straight<br>2. Gay/ Lesbian<br>3. Bisexual

		4. Other
Academic Qualifications	No Academic Qualifications if their response was 2.<br><br>Academic Qualifications typically achieved at age 16 if gave one of qualifications 5-15 and were no longer in full-time education<br><br>Studying for or have attained further academic qualifications if gave one of qualifications 5-15 and were still in full-time education or if gave one of qualifications 1-4.<br><br>Those with foreign qualifications are coded as not answered as we do not have information on what level these qualifications were.	Participants were asked:<br>At what age did you complete your continuous full-time education? If you had a 'gap' year between school and university or college please include it as continuous.<br><br>Have you passed any exams or got any of the qualifications?<br>1. Yes<br>2. No, none<br><br>IF Yes THEN<br>Please read down the list and tell me the highest qualification that you have, that is, the first one you come to.<br>INTERVIEWER: Code one only.<br>1. Degree level qualification<br>2. A-levels<br>3. AS level<br>4. SLC Higher Grade, etc<br>5. O-level, 1975 or earlier<br>6. O-level, after 1975 A-C<br>7. O-level, after 1975 D-E<br>8. GCSE grades A*-C<br>9. GCSE grades D-G<br>10. CSE grade 1, etc<br>11. CSE grades 2-5, etc<br>12. CSE Ungraded<br>13. SLC Lower<br>14. SUPE Lower or Ordinary 15. School Certificate<br>16. Foreign qualification
Socio-Economic Class	Following the Natsal-3 groupings, participants responses were classified as:<br><br>1: Managerial & professional occupations<br>2: Intermediate occupations<br>3: Semi-routine / routine occupations<br>4: Never worked / no job of 10+ hrs/week / not in the last 10 years<br>5: Student in full-time education	Questions asked here were informed by the International Standard occupation codes. For more information, see the International Standard Occupation Codes (SOC):<br>http://www.ons.gov.uk/ons/guide-method/classifications/archived-standard- classifications/standard-occupational-classification-2000/index.html

Limiting Disability	Participants were then grouped in the following way:<br>1: None<br>2: Non-limiting<br>3: Limiting	Participants were asked:<br>"Do you have any long-standing illness, disability or infirmity? By long-standing I mean anything that has troubled you over a period of time, or that is likely to affect you over a period of time?"<br>1. "Yes"<br>2. "No"<br><br>IF Yes THEN<br>"Does this limit your activities in any way?"<br>1. "Yes"<br>2. "No"
Opinion of Own Health	Participants were grouped accordingly.	Respondents were asked: 'How is your health in general?'<br>1: Very good<br>2: Good<br>3: Fair<br>4: Bad<br>5: Very bad
Depression	Respondents could select and record as many conditions as needed. If depression (3) was indicated as one the those treated in the past year then the respondent was classified having 'mentioned' the condition.	Respondents were asked:<br>'In the last 12 months, that is since (date 12 months ago), have you received treatment from a health professional for any of the medical conditions listed on this card?'<br>1. Yes<br>2. No<br><br>IF Yes THEN<br>'Which ones in the last year?'<br>1. Back ache lasting for 3 months or longer<br>2. Any other muscle or bone disease lasting for 3 months or longer<br>3. Depression<br>4. Any other mental health condition<br>5. Any other neurological condition, apart from Parkinson's disease and epilepsy 6 Cancer<br>7. Any thyroid condition<br>8. Any (ovarian/testicular) or pituitary condition<br>9. [WOMEN ONLY] Polycystic ovaries<br>10. [WOMEN ONLY] I have received IVF or other fertility treatment

Quintile of Index of Multiple Deprivation	Respondents were classified:<br>1 (Least Deprived)<br>2<br>3<br>4<br>5 (Most Deprived)	Postcodes were used to obtain IMD scores. The adjusted IMD score was generated using a method by Payne and Abel. The individual country IMD scores combined with the co-efficients and residual values from a linear regression of income and employment on the overall IMD score for each country. The combined scores were generated using the most up-to-date scores for each country at the time. These were IMD 2010 for England, IMD 2011 for Wales, and IMD 2009 for Scotland.
Sexual Function	Participants were classified, though this scale, as either low functioning, normal functioning, or not sexually active. The lowest quintile class for each gender determined the 'low functioning' measure.	Participants responded to the 17-item Natsal-SF scale, which combines measures of issues related to physical function, mental distress, and satisfaction. While some were asked all 17 items, those whom have never been in a relationship or have not been sexually active in the past year answered only those which applied and modeling techniques were used to estimate the missing answers.<br><br>For more information, see Mitchel et al 2011 and Mitchel et al 2012.
Medications in Last Year That Have Limited Sexual Activity or Enjoyment	Participants were grouped accordingly.	Participants were asked:<br><br>Have you taken any medications in the last year that you feel have limited your sexual or enjoyment in any way?<br>1 Yes<br>2 No
Dissatisfied with Sex Life	Participants were classified as 'dissatisfied' with their sex life if their response was 4 or 5.	Participants were asked:<br>I feel satisfied with my sex life<br>1. Agree strongly<br>2. Agree<br>3. Neither agree nor disagree<br>4. Disagree<br>5. Disagree strongly
Distressed/Worried about Sex Life	Participants were classified as 'distressed' or 'worried" about their sex life if their response was 1 or 2.	Participants were asked:<br>I feel distressed or worried about my sex life<br>1. Agree strongly<br>2. Agree<br>3. Neither agree nor disagree

		4. Disagree<br>5. Disagree strongly
Avoided Sex Because of Own or Partner's Sexual Difficulties	Participants were classified as avoiding sex if their response was 1 or 2.	Participants were asked:<br>I have avoided sex because of sexual difficulties, either my own or those of my partner.<br>1. Agree strongly<br>2. Agree<br>3. Neither agree nor disagree<br>4. Disagree<br>5. Disagree strongly
Happy in Relationship	Participants were classified as "happy in relationship" if their response was 1 or 2.	Participants were asked:<br>On a scale of 1 to 7, where 1 means very happy and 7 means very unhappy, how happy or unhappy are you with your relationship with your partner, all things considered? 1..7
STI diagnosis	Respondents who reported an STI diagnosis and specified 1 or 2 for the follow up questions, were recorded as having an STI diagnosis in the past 5 years.	Participants were asked:<br>Have you ever been told by a doctor or other healthcare professional that you had any of the following?<br><br>Please select any that you have had, even if not transmitted by sex (women only: Thrush). If more than one, press the space bar between each number.<br>If you have not had any, please select 'none of these' at the bottom of the list.<br><br>1. Chlamydia<br>2. Gonorrhoea<br>3. Genital warts (venereal warts)<br>4. Syphilis<br>5. Trichomonas vaginalis (Trich, TV)<br>6. Herpes (genital herpes)<br>7. Pubic lice / crabs<br>8. Hepatitis B<br>9. (Men only:) NSU (Non Specific Urethritis), NGU (Non Gonococcal Urethritis)<br>10. (Men only:) Epididymitis<br>11. (Women only:) Pelvic Inflammatory Disease (PID, salpingitis)<br>12. (Women only:) Vaginal thrush (Candida, Yeast infection)

13. (Women only:) Bacterial vaginosis
14. Yes, but can't remember which
15. None of these

Respondents that selected 1-9, or reported a diagnosis of HIV, were then asked to specify "when were you last told by a doctor or healthcare professional that you had ___?"
1. Less than 1 year ago
2. Between 1 and 5 years ago
3. Between 5 and 10 years ago
4. More than 10 years ago

For more information on definitions, phrasings, and other variables consult the Natsal-3 questionnaire and codebook, available at www.natsal.ac.uk

Appendix Table II. Table 2. Sexual partners, practices, behaviours and opinions of male and female participants in NATSAL-3, by age group, 2012, UK, N = 15162

Class Label	Men (%)				Women (%)			
	< 45 (n = 4060)	45-54 (n = 794)	55 - 64 (n = 772)	65 - 74 (n = 667)	< 45 (n = 5842)	45-54 (n = 1123)	55 - 64 (n = 1030)	65 - 74 (n = 874)
Number of partners in last year								
0-1	72	82	82	86	78	89	93	91
2+	26	14	10	4	19	6	2	1
Partners								
Had a concurrent partnership in the last 5 years	18	14	9	4	13	6	2	0.6
Has at least one new partner in past year	35	16	13	5	27	12	5	2
Used internet to find sexual partner in last year *	6	4	3	2	3	2	2	1
Number of occasions of sex § in last 4 weeks								
0	30	29	42	59	27	36	59	75
1-2	18	22	20	16	18	21	14	11
3-4	15	17	12	7	16	15	11	3
5+	32	25	14	7	30	18	9	2
Sexual Practices								
Vaginal sex in last year	84	83	70	52	84	79	57	34
Given or received oral sex in past year	76	69	50	28	74	60	34	17
Anal sex in past year	17	13	7	3	15	7	3	3
Genital contact without intercourse in past year	72	64	53	34	73	61	39	26
Masturbation								
Masturbated in last month	75	62	49	30	39	36	18	9
Risky Sexual Behaviours								
Had unsafe sex § in past year †	6	7	5	2	6	3	1	0.5
Paid for sex § in last year	1	1	2	0.5	0.0	0.1	0.0	0.1
STI Diagnosis								

Diagnosed with any STI in the last five years	6	2	0.4	0.6	7	1.2	0.3	0.3
Opinion on Sex Life								
Happy in relationship	38	43	35	28	38	39	29	15
Dissatisfied with sex life	16	18	18	17	12	16	15	10
Worried about sex life	9	10	13	10	11	12	9	7
Avoided sex because of own or partner's sexual difficulties	9	12	16	20	10	15	20	16
Sexual Function								
Normal function	71	70	51	40	71	64	45	29
Low function	15	16	20	15	15	19	17	9
Not sexually active	9	11	20	35	9	13	34	52

All numbers are weighted. All participants (denominators vary across variables because of item non-response). Vaginal sex is defined as a man's penis in a woman's vagina. Oral Sex is defined as mouth on a partner's genital area. Anal sex is defined as man's penis in a partner's anus. *If at least one partner ever. †Unsafe defined as two or more partners and no condom use in the last year. §Heterosexual/same-sex vaginal, oral or anal sex. ‡Excluding Thrush

We determined the optimal number of classes based on Akaike Information Criteria (AIC), Bayesian Information Criteria (BIC), and the negative log-likelihood (Appendix Table III). The BIC and AIC are both commonly used in assessing model fit. The main difference between the two is that AIC looks to select the model that has high dimensional reality whereas BIC looks to find the true model among the set of candidates. Thus, BIC is considered to be a better measure than AIC. Negative log-likelihood is related to both measures. For all three, a smaller value indicates a better model fit. In our study, all three measures suggested four classes were the best fit for both men and women.

Appendix Table III: Latent class analyses Model Fit Statistics for Participants Aged 45 and above, N = 4540

	Number of Classes	AIC	BIC	Negative Log-Likelihood
Men	2	12,835.0	12,973.6	6,392.52
	3	12,249.1	12,459.7	6,086.53
	**4**	**11,803.6**	**12,086.3**	**5,850.81**
	5	11,766.2	12,121.0	5,819.08
Women	2	14,840.3	14,987.4	7,395.17
	3	14,139.8	14,363.4	7,031.89
	**4**	**13,842.1**	**14,142.2**	**6,870.04**

5	13,804.3	14,180.9	6,838.16

Figures in bold show that the fit for the four-class model was selected for both men and women. AIC Alkaline Information Criterion, BIC Bayesian Information Criterion, -LogLikelihood Negative log-likelihood

Appendix Table IV. Socio-demographic, health and lifestyle characteristics of male participants, N = 1887

Class Label	Class 1 Content Caseys (%) (n = 913)	Class 2 Occasional Ollies (%) (n = 583)	Class 3 Low Function Frankies (%) (n = 218)	Class 4 Multiple-Partner Morgans (%) (n = 173)	P-Value
Age Group					< .0001
45 - 54	44	23	38	53	
55 - 64	33	32	38	36	

65 - 74	23	46	25	11	
Ethnicity					< .0001
White	95	96	94	93	
Mixed	1	1	1	1	
Asian	3	2	3	2	
Black	2	0	2	4	
Other	0	0	0	1	
Relationship Status					< .0001
Married or civil partnership	74	37	73	25	
Living with a partner	10	4	10	5	
Steady relationship, not cohabiting	12	1	6	23	
No steady relationship	5	58	11	46	
Education					< .0001
No academic qualifications	27	46	29	34	
Academic qualifications typically gained at age 16 years †	35	22	31	34	
Studying for or have attained further academic qualifications	37	31	39	32	
Quintile of Index of Multiple Deprivation					< .0001
1 (least deprived)	25	18	26	19	
2	25	22	19	21	
3	20	20	20	19	
4	16	20	15	19	
5 (most deprived)	14	19	19	22	
Sought help or advice for sex life in the past year					< .0001
Yes	8	10	34	13	
No	92	90	66	87	
Currently taking medicine prescribed by a doctor for depression					< .0001
Yes	3	8	10	7	
No	97	92	90	93	
Medications in last year that have limited sexual activity or enjoyment					< .0001

Yes	9	18	28	11	
No	91	79	71	89	
Respondent's opinion of own health					< .0001
Very Good	38	19	21	33	
Good	44	39	42	41	
Fair	14	29	29	20	
Bad	3	10	6	6	
Very Bad	0	3	2	1	
Respondent has a longstanding illness, disability or infirmity					< .0001
None	60	40	44	62	
Non-Limiting	22	22	21	15	
Limiting	19	37	35	23	

All estimates are unweighted. Probabilities greater than 50% are bolded to indicate items that members of a given class were more likely to endorse. †English General Certificate of Secondary Education or equivalent.

Appendix Table V. Socio-demographic, health and lifestyle characteristics of female participants, N = 2653

Class Label		Class 1	Class 2	Class 3	Class 4	P-Value
		Content Caseys (%)	Infrequent Indigos (%)	Low Functioning Lees (%)	Multiple-Partner Morgans (%)	
		(n = 2158)	(n = 1727)	(n = 424)	(n = 231)	*p*
Age Group						
	45 - 54	49	19	54	64	
	55 - 64	34	37	33	29	
	65 - 74	17	44	13	7	< 0.0001
Ethnicity						
	White	94	95	94	92	
	Mixed	0.9	0.6	0.9	1	
	Asian	3	2	3	1	
	Black	2	2	2	5	
	Other	0.5	0.7	0.2	0.9	0.12
Relationship Status						
	Married or civil partnership	72	34	71	26	
	Living with a partner	9	3	9	5	
	Steady relationship, not cohabiting	13	0.7	7	22	
	No steady relationship	7	63	13	48	< 0.0001
Education						
	No academic qualifications	28	43	27	33	
	Academic qualifications typically gained at age 16 years †	37	28	34	37	
	Studying for or have attained further academic qualifications	34	27	38	30	<0 .0001
Quintile of Index of Multiple Deprivation						
	1 (least deprived)	26	19	25	20	
	2	25	22	19	21	
	3	19	21	19	19	

4	16	19	17	20	
5 (most deprived)	14	20	20	20	< 0.0001
Sought help or advice for sex life in the past year					
Yes	7	7	32	13	
No	93	94	68	87	< 0.0001
Currently taking medicine prescribed by a doctor for depression					
Yes	5	10	13	10	
No	95	90	87	90	< 0.0001
Medications in last year that have limited sexual activity or enjoyment					
Yes	7	9	23	11	
No	93	88	77	89	< .0001
Respondent's opinion of own health					
Very Good	37	25	25	31	
Good	45	38	41	42	
Fair	15	26	26	20	
Bad	4	9	7	6	
Very Bad	0.6	3	2	0.9	< 0.0001
Respondent has a longstanding illness, disability or infirmity					
None	59	44	46	60	
Non-Limiting	20	21	20	16	
Limiting	21	35	34	25	< 0.0001

All estimates are unweighted. Probabilities greater than 50% are bolded to indicate items that members of a given class were more likely to endorse. †English General Certificate of Secondary Education or equivalent.
