# Supplementary material for: Sexual behaviours and sexual health among middle-aged and older adults in Britain
Source: Sex Transm Infect. 2022 Aug 11;99(3):173–9. doi: 10.1136/sextrans-2021-055346 (PMC10176408; doi:10.1136/sextrans-2021-055346)
Supplement: Abstract translation [file sextrans-2021-055346supp002.pdf]

研究目标
在当代老龄化社会中，我们普遍缺乏具有人群代表性的针对中老年人群性健康的研究。本研究旨在探索 45-74 岁人群潜在的性行为和性健康模式与类型。
研究方法
对英国 2011 年开展的第三次国家性生活方式和态度的全人群调查数据（Natsal-3）、采用潜在类别分析（Latent class analysis）方法进行分析。
研究结果
在 5260 名 45-74 岁的受访者中，48.86%的男性和 44.91%的女性属于‘性健康状况良好’的类别；另外一个类别（30.94%的男性，44.38%的女性）的典型特征是‘缺乏性活动’，同时性满意度较差且更可能有身体失能。11.65%的男性和 8.41%的女性属于‘性功能障碍’类别，其中有更多人自报有身体失能，且有与性功能障碍相关的精神压力。少部分人（8.62%的男性，2.30%的女性）的特点是有多个的性伴侣和高危性行为。
结论
在中老年人群中鉴别这四个类别可以帮助我们为该人群提供个性化性健康服务，以改善其性功能、性满意度，减少中老年人群性健康相关的精神压力和高危性行为，此类服务均应该将失能中老年人群考虑进来。
